# Supplementary material for: Reconciling Mining with the Conservation of Cave Biodiversity: A Quantitative Baseline to Help Establish Conservation Priorities
Source: PLoS One. 2016 Dec 20;11(12):e0168348. doi: 10.1371/journal.pone.0168348 (PMC5173368; doi:10.1371/journal.pone.0168348)
Supplement: S1 Dataset — (ZIP) [file pone.0168348.s002.zip › Taxa/Serra Norte/SN_2013/N5S/Lista N5S-68.pdf]

CAVIDADE N5S-0068

Classe	Ordem	Fam/Outros	Gên/Outros	Espécie	S-U	U-S	Zon.
Annelida	Oligochaeta			jovem		7	E
Arachnida	Acari	Ixodidae		jovem	X		E
Arachnida	Acari	Rhagidiidae		sp.20	X		E
Arachnida	Amblypygi	Phrynidae	*Heterophrynus*	*longicornis*	1		E
Arachnida	Araneae	Corinnidae		jovem	1		E
Arachnida	Araneae	Ctenidae		jovem	3	1	E
Arachnida	Araneae	Linyphiidae	*Meioneta*	sp.1	X		E
Arachnida	Araneae	Mysmenidae	*Microdipoena*	sp.1		X	E
Arachnida	Araneae	Ochyroceratidae	*Ochyrocera*	sp.3	X	X	E
Arachnida	Araneae	Oonopidae	aff. *Xyccarph*	sp.3	X	X	E
Arachnida	Araneae	Oonopidae	*Capitato*	sp.1	X		E
Arachnida	Araneae	Pholcidae	*Mesabolivar*	sp.1	X	X	E
Arachnida	Araneae	Pisauridae		jovem	1	1	E
Arachnida	Araneae	Salticidae		jovem		X	E
Arachnida	Araneae	Tetragnathidae		jovem		X	E
Arachnida	Araneae	Theridiidae	*Argyrodes*	sp.1		X	E
Arachnida	Araneae	Theridiidae	*Theridion*	sp.1	X		E
Arachnida	Araneae	Theridiidae	*Theridion*	sp.2		X	E
Arachnida	Araneae	Theridiosomatidae	*Plato*	sp.1		X	E
Arachnida	Araneae	Theridiosomatidae	*Plato*	sp.2	X		E
Arachnida	Opiliones	Sclerosomatidae	*Prionostema*	sp.1	1		E
Arachnida	Opiliones	Stygnidae	*Protimesius*	*gracilis*		1	E
Arachnida	Pseudoscorpiones	Bochicidae		jovem		X	E
Arachnida	Pseudoscorpiones	Chthoniidae		jovem	X		E
Chilopoda	Scolopendromorpha	Scolopocryptopidae	*Newportia*	sp.1		5	E
Diplopoda	Polydesmida	Chelodesmidae		jovem	1	4	E
Diplopoda	Polydesmida	Fuhrmannodesmidae		jovem		X	E
Diplopoda	Polydesmida	Pyrgodesmidae		sp.1	X		E
Diplopoda	Spirostreptida	Pseudonannolenidae	*Pseudonannolene*	jovem		1	E
Entognatha	Collembola	Entomobryidae		jovem	X		E
Entognatha	Collembola	Paronellidae		sp.1		X	E
Entognatha	Diplura	Campodeidae		sp.1	X	X	E
Entognatha	Diplura	Japygidae		sp.3		X	E
Insecta	Blattodea	Blattellidae		sp.1	1	1	E
Insecta	Coleoptera	Dytiscidae		sp.7		X	E
Insecta	Coleoptera	Dytiscidae		sp.12B		X	
Insecta	Coleoptera	Scydmaenidae		sp.10	X		E
Insecta	Coleoptera	Staphylinidae		sp.2		X	E
Insecta	Diptera	Cecidomyiidae	Cecidomyiinae	sp.		X	E
Insecta	Diptera	Ceratopogonidae		sp.	X		E
Insecta	Diptera	Chironomidae		sp.		X	E
Insecta	Diptera	Psychodidae	Phlebotominae	sp.	X	X	E
Insecta	Diptera	Tipulidae	Tipulinae	sp.	X	X	E
Insecta	Heteroptera	Reduviidae	Emesinae	sp.		1	E
Insecta	Homoptera	Cixiidae		sp.3	X	X	E
Insecta	Hymenoptera	Diapriidae		sp.1	X		E
Insecta	Hymenoptera	Formicidae	*Brachymyrmex*	sp.1	X		E
Insecta	Hymenoptera	Formicidae	*Camponotus*	sp.2	X		E
Insecta	Hymenoptera	Formicidae	*Crematogaster*	sp.1	X	X	E

Insecta	Hymenoptera	Formicidae	*Eurhopalothrix*	sp.1		X	E
Insecta	Hymenoptera	Formicidae	*Gnamptogenys*	sp.1	X		E
Insecta	Hymenoptera	Formicidae	*Hypoponera*	sp.1		X	E
Insecta	Hymenoptera	Formicidae	*Pachycondyla*	sp.2	X		E
Insecta	Hymenoptera	Formicidae	*Paratrechina*	sp.1		X	E
Insecta	Hymenoptera	Formicidae	*Paratrechina*	sp.2		X	E
Insecta	Hymenoptera	Formicidae	*Pheidole*	sp.1		X	E
Insecta	Hymenoptera	Formicidae	*Pheidole*	sp.3	X		E
Insecta	Hymenoptera	Formicidae	*Solenopsis*	sp.1	X		E
Insecta	Isoptera	Termitidae	*Nasutitermes*	sp.	X	X	E
Insecta	Lepidoptera	Noctuoidea		sp.5		1	E
Insecta	Orthoptera	Phalangopsidae	*Paraclodes*	sp.	5	7	E
Insecta	Psocoptera			jovem		X	E
Insecta	Thysanura	Nicoletiidae		sp.1		X	E
Gastropoda	Pulmonata	Subulinidae		sp.5	X		E
Gastropoda	Pulmonata	Systrophiidae	*Happia*	sp.	X	X	E
Malacostraca	Isopoda	Philosciidae		sp.1		X	E
Malacostraca	Isopoda	Philosciidae		sp.2		X	E
Amphibia	Anura	Leptodactylidae	*Leptodactylus*	cf. *vastus*	2		E
Amphibia	Anura	Strabomantidae	*Pristimantis*	cf. *fenestratus*	5	1	E
Reptilia	Squamata	Sauria		sp.	1		E
